# Controlling arbovirus infection: high-throughput transcriptome and proteome insights

**DOI:** 10.3389/fmicb.2024.1330303

**Published:** 2024-02-13

**Authors:** Mireia Puig-Torrents, Juana Díez

**Affiliations:** Molecular Virology Group, Department of Medicine and Life Sciences, Universitat Pompeu Fabra, Barcelona, Spain

**Keywords:** arbovirus, vector-borne diseases, mosquitoes, transcriptome, proteome, vector

## Abstract

Arboviruses pose a significant threat to public health globally, demanding innovative approaches for their control. For this, a better understanding of the complex web of interactions established in arbovirus-infected mosquitoes is fundamental. High-throughput analyses allow a genome-wide view of arbovirus-induced alterations at different gene expression levels. This review provides a comprehensive perspective into the current literature in transcriptome and proteome landscapes in mosquitoes infected with arboviruses. It also proposes a coordinated research effort to define the critical nodes that determine arbovirus infection and transmission.

## Introduction

**FIGURE 1:**
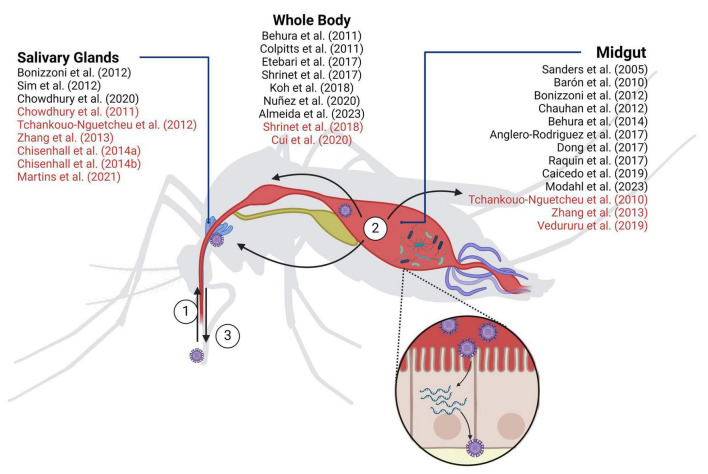
Mosquito-borne virus infection in *Aedes aegypti*. (1) Acquisition: viruses enter the mosquito by ingesting infected blood; (2) Dissemination: infected blood journeys to the midgut for digestion. Here, viruses must successfully infect the epithelial cells of the midgut lining to initiate infection and subsequent systemic dissemination; (3) Transmission: once viruses infect the salivary glands, they are intradermally injected together with the mosquito saliva when the mosquito bites a new host. *In vivo* transcriptomic (black) and proteomic (red) studies are indicated. Created with Biorender.com.

Arboviruses are viruses transmitted to humans and other vertebrates through blood-feeding arthropods. Out of the over 500 identified arboviruses around 70 infect humans causing a broad range of diseases whose symptoms vary from mild to life-threatening. Transmission of these viruses to humans or other vertebrates occurs through the bite of blood-feeding arthropods such as mosquitoes, ticks, sandflies, and midgets. Most human-infecting arboviruses belong to the *Togaviridae*, *Flaviviridae*, or *Bunyaviridae* families, all of which are RNA viruses categorized as class IV or class V in the Baltimore classification. More than 70% of human arboviruses are transmitted by mosquitoes (calculated from Young, 2023). The life cycle of arboviruses in mosquitoes is divided into three main stages: acquisition, dissemination, and transmission (Figure 1). Viruses are acquired by mosquitoes when they feed on infectious blood, which is ingested into the midgut for digestion. To spread throughout the mosquito’s body, viruses need first to efficiently infect the epithelial cells of the midgut linen to establish a preliminary stage of infection. This requires overcoming the midgut barrier formed by the mosquito immune system as well as microbiota that protects the epithelial cells from infection. If the virus successfully breaches this barrier, the new progeny viruses then disseminate into secondary tissues to establish a chronic systemic infection. For transmission to a new host, viruses must infect the salivary glands, allowing them to be intradermally injected into a new host together with mosquito saliva proteins (Franz et al., 2015; Barillas-Mury et al., 2022). Along the different life cycle stages, arboviruses establish a dynamic web of interactions with the infected host that require genome-wide approaches to grasp their complexity. This review provides a comprehensive perspective into the arbovirus-induced alterations of the mosquito transcriptome and proteome landscapes highlighting their major findings and limitations.

The most relevant arbovirus-transmitting mosquito species are included within the genera *Aedes* and *Culex*. Among the species within the *Aedes* genus, *A. albopictus* and *A. aegypti* are particularly noteworthy. They serve as vectors for a range of viruses, including chikungunya virus (CHIKV), dengue virus (DENV), and zika virus (ZIKV). Notably, *A. aegypti* is also a vector for yellow fever virus (YFV). The *A. aegypti* species originated in Africa and spread to the Americas between the 15th and 18th centuries due to slave trade ships, and to Asian countries in the 19th century (Brown et al., 2014). *A. albopictus*, originally from Asian forests, spread to many islands in the Pacific and Indian oceans, and further to Europe (Medlock et al., 2012), and America (Carvalho et al., 2014) around the 1980s. Both species used to feed mainly on animal blood before their expansion to other geographical areas. However, currently they both feed preferentially on human blood (Tempelis et al., 1970; Kamgang et al., 2012; Sivan et al., 2015). Predicted distribution of *Aedes* main vector species show that they are now present in all continents and rapidly expanding to new geographical areas (Kraemer et al., 2015). Among the *Culex* genus, *C. pipiens* is the main vector of West Nile virus (WNV), Eastern equine encephalitis virus (EEEV), Western equine encephalitis virus (WEEV), St. Louis encephalitis virus (SLEV), Sindbis virus (SINV), and Usutu virus (USUV). *C. pipiens* is native of North Africa, West Asia and Europe. Similarly to *Aedes*, due to ships and worldwide movement of the population, nowadays they are distributed worldwide (Haba and McBride, 2022). Most species of *Culex* mosquitoes feed both on human host and other vertebrates. *C. pipiens* predominantly feeds on birds’ blood (42.5–70.5%), playing an important role in the spread of WNV from secondary reservoirs in the avian-to-mammal cycle (Sawabe et al., 2010; Osório et al., 2012; Tiron et al., 2021).

Within the arbovirus group, the DENV, ZIKV, CHIKV, YFV, and WNV viruses are of major public health importance. Together, they cause millions of severe cases worldwide with significant morbidity and mortality rates (Table 1). From these, DENV causes the highest number of infections. Each year, around 400 million people are infected, 100 million develop symptoms and 40.000 die from severe dengue (CDC – Dengue, 2023). These alarming numbers are due to the continuous spread of arboviruses over the last decades out of their endemic tropical and sub-tropical regions into new geographical areas. Indeed, current models estimate that more than 80% of the population within the five continents is currently at risk of at least one vector-borne disease (Weaver and Reisen, 2010; Franklinos et al., 2019). Moreover, viruses like YFV are not only emerging in new areas, but also re-emerging in previously eradicated regions (WHO – Yellow Fever News, 2016). The expansion of arboviruses has been driven by three inherent features of our modern world: global warming, extensive urbanization, and international travel. First, the rise in global temperatures enabled the expansion of mosquito populations into regions that were formerly too cold to support their presence. Moreover, warmer temperatures prolong the mosquito breeding season, resulting in extended periods of potential disease transmission. These elevated temperatures also impact virus susceptibility and the incubation period within mosquitoes (Bellone and Failloux, 2020). Second, the large influx of people into urban areas creates conducive environments for mosquito breeding and disease transmission. These include poorly managed drainage systems in cities that collect and retain stagnant water, along with rainwater accumulation in houses and sewage tanks, which provide ideal breeding grounds (Kolimenakis et al., 2021). Moreover, high population density increases the likelihood of encountering new hosts. Lastly, the surge in long-distance travel facilitates the arrival of infected people who, when bitten by local mosquitoes, can spread the disease to naïve populations. On top of this, public and private transportation systems and their cargos may inadvertently transport mosquitoes or mosquito eggs, introducing both the mosquito vectors and the associated diseases into new regions (Tatem et al., 2012). Overall, the interplay between these factors has created the perfect scenario for the current situation. A paradigmatic example is the recent expansion of ZIKV to the American continent. ZIKV was mainly localized in African countries until 2007 when it caused an outbreak in Micronesia that affected 73% of the Yap Island population (Armstrong et al., 2017). From there, in 2013 ZIKV rapidly spread to the French Polynesia islands and to other Pacific islands (Kucharski et al., 2016), and in 2013–2014 to South America where in 2015 caused a vast epidemic. In 2016, 31 countries reported local transmission of ZIKV (Vest, 2016), resulting in the largest outbreak of the disease with around 700.000 global cases (PAHO/WHO Data Zika weekly report, 2023).

**TABLE 1:** Characteristics of five major human-infecting arboviruses.

Virus	Family	Genome	Cases per year	Symptoms	Mortality rate of severe cases	% Asymp-tomatic	Main mosquito species
Dengue virus	*Flaviviridae*	(+)ssRNA	400 million^1^	*Mild*–fever, nausea, vomits, rash, joint pain	1–20%^2^	75%^3^	*A. aegypti* *A. albopictus*
				*Severe*–vomiting blood, restlessness, shock, internal bleeding, death			
Zika virus	*Flaviviridae*	(+)ssRNA	700.000*^4^	*Mild*–fever, rash, headache, joint pain	<1% (adults)^5^	80%^6^	*A. aegypti* *A. albopictus*
				*Severe*–Guillain-Barré syndrome, microcephaly, miscarriage, stillbirth			
Yellow fever virus	*Flaviviridae*	(+)ssRNA	200.000^7^	*1st phase*–fever, muscle pain, headache, nausea, vomit, shivers, loss of appetite	47%^8^	55%^8^	*A. aegypti* *A. albopictus*
				*2nd phase*–high fever, yellow skin, bleeding, shock, organ failure, death			
West Nile virus	*Flaviviridae*	(+)ssRNA	2.500^9^	*Mild*–fever, headache, body ache, joint pain, vomit, diarrhea, rash	10%^10^	80%^10^	*C. pipiens*
				*Severe*–encephalitis, meningitis, neck stiffness, disorientation, coma, convulsions, paralysis			
Chikungunya virus	*Togaviridae*	(+)ssRNA	440.000^11^	Fever, joint pain, headache, nausea, rash, chronic arthritis	<1%^11^	15%^12^	*A. aegypti* *A. albopictus*

*During the last pandemic (2016).

^1^CDC – Dengue (2023).

^2^ Chagas et al. (2022).

^3^ CDC – Dengue Symptoms (2023).

^4^ PAHO/WHO Data (2022).

^5^ Cardona-Ospina et al. (2019).

^6^ Oster et al. (2016).

^7^ WHO – Yellow Fever (2023).

^8^ Johansson et al. (2014).

^9^ Ronca et al. (2021).

^10^ CDC – Dengue Treatment (2023).

^11^ CDC – Chikungunya (2023).

^12^ Burt et al. (2017).

This dismay scenario stresses the need for better control of arbovirus infections. Currently, there is no specific antiviral therapy for any human arbovirus and only for some of them are vaccines available. Infection by multiple arboviruses, such as DENV, ZIKV, or CHIKV, causes similar initial symptoms, typically including fever, nausea, vomiting, headaches, rash, joint and muscle pain. Without efficient treatments, healthcare professionals have no other options than to advise rest, drinking of fluids and over-the-counter drugs to treat the symptoms. Following the initial phase of infection, a portion of patients develop more severe symptoms (Table 1) and hospitalization might be required for frequent monitoring and intensive care such as intravenous fluids and stronger pain medication (CDC – Chikungunya, 2023; CDC – Dengue Treatment, 2023; CDC – Zika, 2023). To prevent infections, currently there are few approved vaccines: 17D for YFV, Ixiaro (Valneva) for Japanese encephalitis virus (JEV), Dengvaxia (Sanofi) and Qdenga (Takeda) for DENV, and Ixchiq (Valneva) for CHIKV. All are attenuated or inactivated vaccines. The YFV vaccine (17D) was the first arbovirus vaccine proven to be effective since the late 1930s. However, YFV disease has not been eradicated and outbreaks are frequent, likely due to the lack of an extensive vaccination program or insufficient doses (WHO – Yellow Fever News, 2016). Ixiaro is a vaccine approved since 2009 by FDA to prevent Japanese encephalitis. Despite the 98% efficacy of Ixiaro, serious outbreaks still occur, especially in western Pacific countries and in northern Australia (Firbas and Jilma, 2015; Wang and Liang, 2015). Dengvaxia was the first approved vaccine for dengue fever. It consists of the YVF vaccine backbone where the gene prM/E was swapped by the corresponding proteins of each one of the four DENV serotypes. Even though it was approved as a universal DENV vaccine, its efficacy against DENV-2, the serotype causing the highest number of world cases, was shown to be low or non-significant in several phase II and phase III trials (Wallace et al., 2012; Capeding and Tran, 2014; Villar et al., 2015). In addition, a study conducted by SAGE (Strategic Advisory Group of Experts on Immunization of the World Health Organization) evidenced long-term safety issues for seronegative individuals and recommended that only those that had been previously infected with dengue should get vaccinated (WHO, 2017). Qdenga, the other DENV vaccine, was constructed on an attenuated DENV-2 backbone by swapping the prM/E genes of the other serotypes. It has been shown efficient against all four dengue serotypes and safe for both seronegative and seropositive, but currently is only approved in Europe, Thailand and Brazil. Finally, the most recent arbovirus approved vaccine is Ixchiq, the first CHIKV vaccine available (FDA News, 2023). Given the severe disease and prolonged health problems of CHIKV disease, Ixchiq was approved in November 2023 following the accelerated approval pathway from FDA. To improve this scenario, further efforts are being done to find novel vaccine strategies (Silva et al., 2018). Unfortunately, potential cross-reactive antibodies (reviewed by Rathore and St. John, 2020) and antibody-dependent enhancement of viral infection (reviewed by Kulkarni, 2020 and van Bree et al., 2023) influences vaccine safety in areas endemic for more than one virus and strain.

Given the continuous spread of arboviruses, it is increasingly important to interrupt the transmission of diseases by arthropod vectors in order to combat these diseases effectively. Conventional approaches to vector control, like focusing on the elimination of breeding sites and the use of insecticides, have offered only restricted efficacy in reducing the spread of infections (Morrison et al., 2008). Moreover, the use of insecticides causes serious environmental problems and poses a strong selective pressure that might result in resistance (Ranson et al., 2011; Moyes et al., 2017). Indeed, insecticide resistance within native *A. aegypti* populations is currently widespread on a global scale (IR Mapper, 2023). In recent years, scientific progress has paved the way for innovative vector control strategies that use living organisms to reduce arbovirus transmission. The so-called biological control methods encompass introducing the symbiotic proteobacteria *Wolbachia* into *A. aegypti* or the generation of genetically modified mosquitoes to suppress the mosquito population or to decrease their susceptibility to arboviruses.

Population suppression strategies consist of releasing male mosquitoes incapable of producing offspring. This strategy operates on the premise that a decrease in the mosquito population will result in reduced virus transmission. There are two primary methodologies employed to achieve this objective: the Sterile Insect Technique (SIT) and the Incompatible Insect Technique (IIT) with *Wolbachia* bacteria (Flores and O’Neill, 2018). Under the SIT, male mosquitoes undergo irradiation with x-rays or are treated with sterilizing chemicals, causing chromosomal aberrations or lethal mutations in their sperm. After release, these modified males compete with their wild counterparts in mating with wild females, thereby leading to a reduction in the wild mosquito population. In contrast, the IIT-Wolbachia method involves introducing a strain of the intracellular bacteria *Wolbachia*, which is not naturally found in *A. aegypti*, into the mosquito. This introduction results in the production of inviable offspring due to cytoplasmic incompatibility (CI) when infected males mate with healthy females. However, when female mosquitoes are infected with *Wolbachia*, and mate with either infected or non-infected males, CI does not occur (Chen et al., 2020). By releasing only male mosquitoes infected with *Wolbachia*, mating with wild females fails to produce viable offspring, effectively reducing the mosquito population. While these methods have proven useful, they have the potential to disrupt ecological trophic chains and offer only temporary protection against mosquito-borne viruses, as migration of wild mosquitoes into the controlled area can perpetuate the problem.

Population replacement strategies aim to transmit traits to mosquitoes that impede their infection by arboviruses. With this strategy, after replacing the target population, subsequent releases would be unnecessary or needed only intermittently. The methodologies employed usually release both females and males which would mate with wild type mosquitoes, creating resistant offspring and ideally avoiding the need for recurrent re-insertion into the environment. One of the most encouraging population replacement method involves substituting native *A. aegypti* populations, which are highly susceptible to DENV, CHIKV, and ZIKV, with a *Wolbachia*-transinfected mosquito strain that diminishes the vector competency for these arboviruses (Moreira et al., 2009). The World Mosquito Program has been implementing this method in thirteen countries in Latin America, Asia, and Oceania with excellent results in Australia and Indonesia (World Mosquito Program, 2023). However, this has not been the case in Brazil and other geographical areas (Ribeiro, dos Santos et al., 2022). A primary drawback of the *Wolbachia* strategy for controlling the transmission of arboviruses by *A. aegypti* is its reliance on specific local factors, such as climate, environmental conditions, geographical barriers, and genetic compatibility between native and released mosquitoes (Schmidt et al., 2017; Pavan et al., 2023). This makes the strategy less universally applicable and limits its effectiveness in certain regions. Furthermore, there is a significant gap in our understanding of the mechanisms behind the protection conferred by *Wolbachia* against arboviruses. This lack of knowledge complicates our ability to track or prevent potential mutant viruses from evading this protection (Yen and Failloux, 2020). Given these challenges, it becomes crucial to develop alternative strategies for generating arbovirus-resistant mosquitoes. Great advances in genetic manipulation can now be applied to replace arbovirus-transmitting mosquitoes with transgenic arbovirus-resistant ones (Powell, 2022). The CRISPR-Cas9 technology allows targeting specific places in the genome and gene-driving strategies, such as CRISPR-Cas9-directed homologous repair, to shift naturally occurring allele frequencies and spread the genetic manipulation throughout the mosquito population (Alphey, 2016). However, despite decades of research on arbovirus-mosquito vector interactions, the identification of a genetic target that inhibits arbovirus infection without affecting mosquito fitness remains elusive (Powell, 2022). Deciphering such targets is key to developing new biological control and to improve our understanding of vector biology. To identify these targets effectively, it is crucial to enhance our understanding of the intricate web of interactions established by arboviruses within infected mosquitoes. High-throughput analyses, such as transcriptomics, translatomics, and proteomics are invaluable tools to unravel such interactions in an unbiased manner. Notably, the impact of arboviruses on the mosquito’s translation landscape remains largely unexplored. Nevertheless, numerous studies have examined how arboviruses alter host mRNA levels, by RNA-seq and microarray analysis, and host protein expression, by mass spectrometry techniques.

## Genome-wide transcriptome approaches to elucidate mosquito-arbovirus interaction networks

Table 2 aims to summarize all genome-wide studies on arbovirus-induced alterations of mosquito’s mRNA levels. Most studies carried out the analysis in *A. aegypti* mosquitoes, the most relevant vector in the context of human arboviruses. It is worth noting, however, that different strains are used across studies, with the Rockefeller strain being the most used. Transcriptomic data is also accessible for *A. albopictus*, *C. pipiens*, and for the three mosquito cell lines Aag2 (Li et al., 2020), U4.4 (Öhlund et al., 2022), Hsu (Paradkar et al., 2015) derived from *A. aegypti*, *A. albopictus*, and *C. quinquefasciatus*, respectively. Among the studied viruses, DENV-2 infection was the most extensively examined, followed by ZIKV and WNV. Furthermore, several studies meticulously dissected transcriptome alterations within distinct mosquito tissues (mainly midgut and salivary glands) (Figure 1) offering a comprehensive knowledge of how viruses impact transcriptomes across all stages of infection. Related to the time post-infection (tpi) to be analyzed, most publications took various infection time-points, spanning from initial stages of less than 24 hours to prolonged chronic periods of more than 1 week. This approach was prompted by the distinct phases of viral dissemination. While variations can exist among different mosquito strains and arboviruses, the initial phase of approximately 24 hours is typically marked by the virus being predominantly constrained within the mosquito’s midgut. Subsequently, between 2 and 5 days after infection, dissemination starts, spreading the infection to other tissues and organs. Lastly, around 1 week post-infection, salivary glands achieve their peak viral load and infection reaches a steady state. An inherent challenge in synthesizing the findings of these analyses, already mentioned by Angleró-Rodríguez et al. (2017) and Vedururu et al. (2019), is the diverse response to infection exhibited by each virus and mosquito strain that often led to contradictory outcomes. Table 2 reports experimental conditions, viruses, mosquito species and strains, tissues and time of infection used in the different studies. These diverse experimental conditions complicate comparative analysis and hamper definitive conclusions regarding potential targets. Nevertheless, we have summarized the main findings of differentially expressed pathways, aiming to facilitate comparisons for better understanding (Table 3).

**TABLE 2:** Transcriptomic studies during arbovirus infection in mosquitoes.

References	Mosquito strain / cell line	Virus	Infection time	Tissue	Method
***Aedes aegypti***					
Sanders et al., 2005	Rexville D (HWE)	SINV	1, 4 and 8 dpf	Midgut	Microarray
Barón et al., 2010	Cali-S and Cali-R	DENV-2	48 hpf	Midgut	SSH
Colpitts et al., 2011	Rockefeller	WNV, DENV-2 and YFV	1, 2 and 7 dpi	Whole body	Microarray
Behura et al., 2011	Moyo-S and Moyo-R	DENV-2	3 and 18 hpi	Whole body	Microarray
Chauhan et al., 2012	Moyo-S and Moyo-R	DENV-2	1, 4, 24, 48 h and 4 dpf	Midgut	Microarray
Sim et al., 2012	Rockefeller/UGAL	DENV-2	14 dpf	Salivary glands and carcasses	Microarray
Bonizzoni et al., 2012	Chetumal	DENV-2	1, 4 and 14 dpi	Midgut, salivary glands and carcass	RNA-seq
Behura et al., 2014	Moyo-S and Moyo-R	DENV-2	3 h and 3 dpf	Midgut	Microarray
Shrinet et al., 2017	NS	DENV and CHIKV	24 hpi	Whole body	RNA-seq
Angleró-Rodríguez et al., 2017	Rockefeller	DENV-2 and ZIKV	7 dpf	Midgut	RNA-seq
Dong et al., 2017	HWE	CHIKV	1 and 2 dpf	Midgut	RNA-seq
Etebari et al., 2017	Galveston	ZIKV	2, 7 and 14 dpi	Whole body	RNA-seq
Raquin et al., 2017	Thailand	DENV-1	1 and 4 dpi	Midgut	RNA-seq
Koh et al., 2018	Cairns	DENV-3	EIP of 6 to 12 dpi	Whole body	RNA-seq
Caicedo et al., 2019	Cali-S and Cali-R	DENV-2	30 hpi	Midgut	Microarray
Chowdhury et al., 2020	Singapore	DENV-2, ZIKV and CHIKV	14 or 7 dpi	Salivary glands	RNA-seq
Li et al., 2020	Aag2	DENV-2	4 dpi	–	RNA-seq
Coatsworth et al., 2021	Cali-S and Cali-R	DENV-2	24, 36 and 48 hpf	Midgut	RNA-seq
Almeida et al., 2023	Rockefeller	ZIKV	7 and 14 dpf	Whole body	RNA-seq
***Aedes albopictus***					
Vedururu et al., 2019	Foshan	CHIKV	2 dpi	Midgut	RNA-seq
Liu et al., 2022	Foshan	DENV-2	7 dpf	Midgut	RNA-seq
Öhlund et al., 2022	U4.4	WNV	24 and 48 hpi	–	RNA-seq
Modahl et al., 2023	Singapore (also *A. malayensis*)	DENV-2 and CHIKV	1 and 4 dpf	Midgut	RNA-seq
***Culex* sp.**					
Paradkar et al., 2015	Hsu	WNV	48 hpi	–	RNA-seq
Núñez et al., 2020	*pipiens* and *molestus* hybrid from Gavà	RVFV	2 h, 3 and 14 dpe	Whole body	RNA-seq

SSH, suppressive subtractive hybridization; HWE, Higgs’ White Eye; NS, not specified; SINV, Sindbis virus; WNV, West Nile virus; DENV, dengue virus; CHIKV, Chikungunya virus; ZIKV, Zika virus; RVFV, Rift Valley fever virus; h, hours; d, days; hpi, hours post-infection; dpi, days post-infection; dpf, days post-feeding; dpe, days post-exposure; NS, not specified; EIP, extrinsic incubation period.

**TABLE 3:** Main ontologies differentially expressed after infection separated by viral infection.

Ontology		DENV		ZIKV		CHIKV		WNV		YFV		RVFV		SINV		MAYV	
		V	C	V	C	V	C	V	C	V	C	V	C	V	C	V	C
Amino acid metabolism	T																
	P																
Anatomical barriers components	T																
	P																
Blood-feeding	T																
	P																
Carbon metabolism	T																
	P																
Cell death	T																
	P																
Cell signaling	T																
	P																
Chaperone proteins	T																
	P																
Cytoskeleton	T																
	P																
Digestive processes	T																
	P																
Immune response	T																
	P																
Ion binding proteins	T																
	P																
Lipid metabolism	T																
	P																
Odor-binding proteins	T																
	P																
Proteasome	T																
	P																
Redox processes	T																
	P																
Transcription	T																
	P																
Translation	T																
	P																
Transport	T																
	P																

T, transcriptome study; P, proteome study; V, *in vivo* study; C, in cell culture study; DENV, dengue virus; ZIKV, zika virus; CHIKV, chikungunya virus; WNV, West Nile virus; YFV, yellow fever virus; RVFV, Rift Valley fever virus; SINV, Sindbis virus; MAYV, Mayaro virus. Green=upregulated; Red=downregulated; Blue=both directions. Data based on publications of Tables 2, 4.

Collectively these studies have yielded crucial insights into our understanding of arbovirus-mosquito interactions. Numerous immunological key factors were highlighted for their role in the intrinsic defense mechanism of mosquitoes against arboviruses. RNA interference (RNAi) is the principal insect innate immune response. This pathway was shown to be upregulated under DENV (Chauhan et al., 2012; Chowdhury et al., 2020; Modahl et al., 2023), ZIKV (Angleró-Rodríguez et al., 2017; Chowdhury et al., 2020) and CHIKV infections (Chowdhury et al., 2020), but downregulated under SINV (Sanders et al., 2005), Rift Valley fever virus (RVFV) (Núñez et al., 2020), and CHIKV (Modahl et al., 2023) infections. The observed opposite results in the two CHIKV studies might be related to the different organ analyzed or the *Aedes* species studied (see Table 2). Other immune response pathways, such as the Toll-like receptors signaling pathway (TLR), the immunodeficiency pathway (IMD) and Janus kinase/signal transduction and transcription activation pathway (JAK/STAT) were also altered under arbovirus infection conditions.

In midgut, DENV infection induced the expression of some of the Toll pathway components and associated effector molecules like c-type lectins (CTLs) (Angleró-Rodríguez et al., 2017), fibrinogen-related proteins (FREPs), antimicrobial peptides (AMPs) (Angleró-Rodríguez et al., 2017; Modahl et al., 2023) and JNK (Modahl et al., 2023). However other components of the TLR pathway were shown to be downregulated (Bonizzoni et al., 2012; Angleró-Rodríguez et al., 2017), together with serine-rich proteases (SRPs) (Angleró-Rodríguez et al., 2017; Raquin et al., 2017) and leucine-rich proteins (LRR) of the MAPK pathway (Raquin et al., 2017). In salivary glands DENV infection induced the downregulation of immune response components such as TLRs, IMDs, AMPs, SRPs, and CTLs in some studies (Bonizzoni et al., 2012; Chowdhury et al., 2020) while other studies described the upregulation of components from the same pathways (Sim et al., 2012; Chowdhury et al., 2020). Additional studies that compare DENV-induced transcriptome changes in DENV-susceptible vs. -refractory *A. aegypti* mosquitoes pondered the question of whether alterations in the expression of transcripts expressing immune response components affect the midgut barrier escape (Barón et al., 2010; Behura et al., 2011, 2014; Chauhan et al., 2012; Caicedo et al., 2019; Coatsworth et al., 2021). Interestingly, TLRs, JAKs, and MAPK signaling components were upregulated in both the susceptible (Behura et al., 2011, 2014; Chauhan et al., 2012) and the refractory strains (Behura et al., 2011, 2014; Chauhan et al., 2012; Coatsworth et al., 2021). However, other factors like SRPs, AMPs, and cathepsin were found to be downregulated in some studies (Caicedo et al., 2019; Coatsworth et al., 2021) and upregulated in another (Behura et al., 2014) in the refractory strain. Different strains, and time points of investigation might explain the observed differences between studies. Regardless, the mosquito antiviral defense is undoubtedly affected during DENV infection (Table 3). ZIKV and CHIKV infections also induce alterations in the expression of components of the immune system. In midgut, Angleró-Rodríguez et al. (2017) showed a ZIKV-induced upregulation of AMPs, LRRs, SRPs, and TLRs, but a downregulation of FREPs, CTLs and the IMD pathway. On the other hand, ZIKV infection of salivary glands induced an upregulation of SRPs, and both up and down regulation of the IMD pathway (Chowdhury et al., 2020). In midgut, CHIKV induced expression of AMPs, FREPs, and IMD components in *A. aegypti* at early times post-infection (Dong et al., 2017) while parallel studies in *A. albopictus* showed an upregulation of TLRs, SRPs and JNKs (Modahl et al., 2023). Moreover, Modahl et al. (2023) showed that in later timepoints the infection induced a different immune response. Other studies focused on WNV (Öhlund et al., 2022), SINV (Sanders et al., 2005), and RVFV infections (Núñez et al., 2020) also showed variances in the specific components of immune pathways to be altered. All in all, these results might indicate a tailored immune response according to mosquito species and arbovirus.

Multiple cellular pathways beyond immune-related ones were shown to be differentially expressed in response to arbovirus infection. For example, Sanders et al. (2005) reported that SINV infection affected expression of genes related to metabolic transport associated proteins, RNA binding and translation, vesicle transport processes and post-translation modifications genes. Another example is Koh et al. (2018) that reported that DENV-3 infection downregulated the expression of genes associated with energy metabolism, defense response and ion transport and enriched ribosome biogenesis and also other ion transport components. Several other studies mentioned the arbovirus-induced upregulation of apoptosis (Barón et al., 2010; Behura et al., 2011, 2014; Bonizzoni et al., 2012; Núñez et al., 2020; Coatsworth et al., 2021) and autophagy (Chauhan et al., 2012; Behura et al., 2014), most likely triggered by the mosquito immune response. Interestingly, multiple independent studies identified an arbovirus-induced downregulation in the expression of genes related to cuticle (ZIKV, Shrinet et al., 2017; DENV-2, Behura et al., 2011; Shrinet et al., 2017) the cuticle-like protein AAEL011045 (WNV, YFV and DENV-2, Colpitts et al., 2011) or chitin-related genes associated with the cuticle rigidity (ZIKV, Etebari et al., 2017; SINV, Sanders et al., 2005; DENV-2 and YFV, Colpitts et al., 2011). Moreover, AAEL011045 overexpression decreased WNV infection both in cell culture and in mice as a consequence of the cuticle-like protein binding to the envelope protein and inhibiting viral entry (Colpitts et al., 2011). It has been proposed that the cuticle components like chitin proteins may have an important role in the midgut anatomical barrier and its downregulation would facilitate viral dissemination. Other key ontologies identified in studies listed on Table 2 are summarized in Table 3.

Multiple studies observed that when different times post-infection are analyzed, there is an absence of correlation among differentially expressed genes between time-points (Sanders et al., 2005; Colpitts et al., 2011; Bonizzoni et al., 2012; Chauhan et al., 2012; Etebari et al., 2017; Raquin et al., 2017; Núñez et al., 2020; Modahl et al., 2023). The highest occurrence of differentially expressed genes is concentrated within the intermediate infection stages, when the virus has overcome the midgut barrier and starts rapid proliferation for tissue invasion (Sanders et al., 2005; Bonizzoni et al., 2012; Etebari et al., 2017; Raquin et al., 2017). This pattern could be anticipated considering that at early phases the infection levels are relatively low and at later stages a steady state of chronicity is attained. Moreover, the strongest modulation at early time points happens in the midgut, where virus and mosquito interactions first occur (Raquin et al., 2017).

The virus ability to infiltrate the midgut, the first barrier to establish infection, and the salivary glands, the barrier for efficient transmission, is a defining factor of vector competence. Transcriptome studies in midgut and salivary glands as separate compartments, brought interesting insights into infection dissemination within these tissues. In the midgut, activation of the immune response was deeply affected by the temperature at which the mosquitoes were reared (Liu et al., 2022). Moreover, ZIKV and DENV-2 infection elicit both conserved and unique responses in the midgut tissue that involve a variety of functional gene groups (Angleró-Rodríguez et al., 2017; Chowdhury et al., 2020). An example of a shared conserved response is the increased expression of mRNA encoding vATPase (Behura et al., 2011; Angleró-Rodríguez et al., 2017). Moreover, this feature is also conserved in YFV and WNV (Colpitts et al., 2011), in whole mosquitoes. Together, this indicates a potential pan-flavivirus infection-responsive gene. Interestingly genes related to the lipid metabolism such as SREBP in DENV-2 infection (Angleró-Rodríguez et al., 2017) and NPC2 in CHIKV (Vedururu et al., 2019) both functionally shown to be required for viral infections, were also upregulated. In the salivary glands, particularly intriguing was the observation that many differentially expressed genes corresponding to genes crucial for viral transmission, such as odorant binding proteins, were upregulated at late time points (Sim et al., 2012; Etebari et al., 2017).

Transcriptomic responses exhibit virus-specific patterns (Colpitts et al., 2011; Angleró-Rodríguez et al., 2017; Etebari et al., 2017; Shrinet et al., 2017). Upon challenging *A. aegypti* with different arboviruses, mostly virus-specific transcriptomic alterations were observed. For instance, a comparison between DENV-2 and ZIKV showed that nearly 60% of altered genes in the midgut were virus-specific, while 35% exhibited regulated expression in the same direction for both viruses. The latter were enriched in key factors of the innate immune system (Angleró-Rodríguez et al., 2017). Colpitts et al. (2011) conducted a comparative analysis of mosquito gene expression in response to three distinct viral infections: DENV-2, YFV, and WNV. They observed a limited overlap in differentially expressed genes between these three viruses, with notable exceptions at the early stages of infection, when virus-mosquito interactions primarily occur in the midgut. An additional study compared DENV-2 and CHIKV with the same mosquito strain and revealed a similar pattern of minimal overlap among differentially expressed genes (Shrinet et al., 2017).

## Proteomics approaches to elucidate mosquito-arbovirus interaction networks

Table 4 aims to summarize published proteome-wide studies on arbovirus-induced alterations of the mosquito’s proteome. While *A. aegypti* is the predominant model used, the strains vary among studies. Proteomic data is also accessible for *A. albopictus* and for three major mosquito cell lines, U4.4 (Kumar et al., 2022) and C6/36 (Patramool et al., 2011; Zhang et al., 2013; Lee and Chu, 2015; Saucereau et al., 2017; Xin et al., 2017) (both derived from *A. albopictus*) and Aag2 (Vasconcellos et al., 2020, 2022) (derived from *A. aegypti*). Among the studied arboviruses, CHIKV, followed by DENV-2 and ZIKV have been the most extensively examined. Furthermore, several publications explored different infection time-points to cover the diverse phases of infections.

**TABLE 4:** Proteomic studies during arbovirus infection in mosquitoes.

References	Mosquito strain / cell line	Virus	Infection time	Tissue	Method
***Aedes aegypti***					
Tchankouo-Nguetcheu et al., 2010	Liverpool	DENV-2 and CHIKV	7 dpf	Midguts	2D-DIGE + MS/MS
Tchankouo-Nguetcheu et al., 2012	PAEA	CHIKV	3 and 5 dpf	Salivary glands	2DE + MS/MS
Chisenhall et al., 2014a	Rockefeller	DENV-2	10 dpi	Saliva	2DE + LC-MS/MS
Chisenhall et al., 2014b	Rockefeller	DENV-2	10 dpi	Salivary glands	2DE + LC-MS/MS
Shrinet et al., 2018	NS	DENV-2 and CHIKV	24 hpi	Whole	LC-MS
Cui et al., 2020	HWE	CHIKV	0.5, 12, 24, 36, 48, and 72 hpf	Whole	LC-MS/MS
Vasconcellos et al., 2020	Aag2	MAYV	12 and 48 hpi	–	LC-MS/MS
Chowdhury et al., 2021	Singapore	DENV-2, ZIKV, CHIKV	14 dpi	Salivary glands	LC-MS/MS
Martins et al., 2021	Rio de Janeiro	ZIKV	14 dpi	Salivary glands and head	LC-MS/MS
Vasconcellos et al., 2022	Aag2	CHIKV	12 and 48 hpi	–	LC-MS/MS
***Aedes albopictus***					
Patramool et al., 2011	C6/36	DENV-1 and DENV-3	48 hpi	–	2D-DIGE + MS
Zhang et al., 2013	Guangzhou and C6/36	DENV-2	8 dpi or 24 hpi	Salivary glands and midgut	2D-DIGE + MS/MS
Lee and Chu, 2015	C6/36	CHIKV	24, 48, and 96 hpi	–	2DE + MS/MS
Xin et al., 2017	C6/36	ZIKV	96 hpi		LC-MS/MS
Saucereau et al., 2017	C6/36	CHIKV	24 hpi	–	2DE + LC-MS/MS
Kumar et al., 2022	U4.4	CHIKV	12 and 60 hpi	–	LC-MS/MS

HWE, Higgs’ white eye; NS, not specified; DENV, dengue virus; CHIKV, Chikungunya virus; ZIKV, Zika virus; MAYV, Mayaro virus; hpi, hours post-infection; dpi, days post-infection; dpf, days post-feeding; MS, mass spectrometry; MS/MS, tandem mass spectrometry; LC, liquid chromatography; 2DE, 2-dimensions electrophoresis; 2D-DIGE, two-dimensional difference gel electrophoresis.

The proteome analyses show that numerous cellular pathways are affected during viral infection, with a predominant pattern of protein downregulation (Chisenhall et al., 2014b; Saucereau et al., 2017; Vasconcellos et al., 2022). Despite variations in research findings between studies, a consensus emerges regarding the substantial alteration of immune-related factors (Tchankouo-Nguetcheu et al., 2010; Chisenhall et al., 2014b; Xin et al., 2017; Vasconcellos et al., 2020), particularly those linked to the Toll-like receptor pathway. TLRs, FREPs and CTLs were mainly downregulated in DENV, ZIKV and CHIKV infection (Chisenhall et al., 2014b; Cui et al., 2020; Chowdhury et al., 2021), except for CTL5 in ZIKV infection, which was upregulated (Chowdhury et al., 2021). Some SRPs were described to be downregulated during DENV infection in saliva (Chisenhall et al., 2014a) while other SRPs were described to be upregulated or downregulated in salivary glands after DENV, CHIKV, and ZIKV infection (Chowdhury et al., 2021). AMPs were also seen to be upregulated in both CHIKV and ZIKV infected mosquitoes (Cui et al., 2020; Martins et al., 2021). Additional alterations observed in some studies affected pathways related to cell metabolism (Tchankouo-Nguetcheu et al., 2010, 2012; Patramool et al., 2011; Zhang et al., 2013; Chisenhall et al., 2014b; Vasconcellos et al., 2020, 2022; Martins et al., 2021; Kumar et al., 2022) such as glycolysis and oxidative stress (Tchankouo-Nguetcheu et al., 2010, 2012; Patramool et al., 2011; Shrinet et al., 2018; Vasconcellos et al., 2020), cytoskeleton (Zhang et al., 2013; Cui et al., 2020), and vesicle transport (Xin et al., 2017; Cui et al., 2020; Martins et al., 2021; Table 3).

Proteome analyses reveal limited correlations between different time-points after infection. Moreover, proteome alterations exhibit virus-specific patterns. Related to the proteome alterations at different time points, CHIKV infection of *A. aegypti* upregulated the expression of proteins related to endocytosis, energy metabolism, ribosome biogenesis and vesicular transport at early times of infection while at late times of infection multiple proteins related to similar pathways were downregulated (Cui et al., 2020). In turn, proteome upregulations induced by CHIKV infection of the Dicer-2-deficient C6/36 cell line at different time points, affected proteins related to metabolic processes, cytoskeleton proteins, folding and DNA synthesis. Typically, this upregulation peaked at 48 hpi and declined at 96 hpi (Lee and Chu, 2015). This peaked upregulation of protein expression was also observed in CHIKV-infected Aag2 cells, however, the pathways involved include amino acid and carbon metabolism (Vasconcellos et al., 2022). Parallel work in U4.4 cell line show upregulation of proteins related to ribosome processing, ribosome biogenesis, translation and gene expression at 60 hpi (Kumar et al., 2022). Although the specific pathways shown to be upregulated *in vivo* and in the different cell lines differ, they all shared their involvement in increasing the synthesis capacity of the cell, a trait envisaged to be needed to establish a chronic infection. Related to the virus-specific alterations of the mosquito proteome three studies carried out parallel analyses of different arbovirus in *A. aegypti* (Tchankouo-Nguetcheu et al., 2010; Shrinet et al., 2018; Chowdhury et al., 2021). The study of Shrinet et al. (2018) focuses in proteome changes induced by DENV and CHIKV at the level of the whole mosquito at 24 hpi. Although, they show some similarities between viruses, they observed that CHIKV infection mainly activated oxidative stress pathways, triggering the immune system, while DENV-2 primarily affected energy metabolism. These differences might be explained by their different infection dynamics. Whereas CHIKV has a fast replication rate, all DENV strains replicate slower. In turn the studies of Tchankouo-Nguetcheu et al. (2010) and Chowdhury et al. (2021) focused in arbovirus-induced proteome changes in specific organs. The study by Chowdhury et al. (2021) in salivary glands shows that the observed alterations elicited by DENV, ZIKV or CHIKV were mostly virus-specific. However, they identified four shared upregulated proteins (SGBAP, SGS1, ADA, and GILT-like). Interestingly SGBAP displayed an antiviral function against all three viruses. The study by Tchankouo-Nguetcheu et al. (2010) in midgut observed that although there were differences between the alterations elicited by CHIKV and DENV, both viruses induce an overexpression of proteins involved in cell protection, particularly in proteins involved in the antioxidant response and in detoxification.

Several studies focus on proteome changes induced by arbovirus infections in specific mosquito tissues. Of special interest are those focused on salivary glands tissues and saliva, as they can offer crucial insights into the transmission of viruses via mosquito bite. Two separate studies highlighted the downregulation of the hemostatic protein apyrase during infection of salivary glands in both CHIKV and DENV-2 infections (Tchankouo-Nguetcheu et al., 2012; Chisenhall et al., 2014a, respectively). Interestingly, other anti-hemostatic and pain inhibitory proteins were also found to be downregulated (Chisenhall et al., 2014a). Moreover, CHIKV infection downregulated the expression of allergen aegyptin protein in other study (Chisenhall et al., 2014b), potentially reducing inflammation at the biting sites. Together, these results uncover complex interactions at mosquito bite locations that promote arbovirus infection. Downregulation of anti-hemostatic proteins in the mosquito’s saliva increases the probability of blood clotting while feeding. This would result in an increase in biting rates. Similarly, downregulation of pain inhibitory proteins would alert the human host, causing an interruption of the feeding and the need of the mosquito to feed on other hosts (Chisenhall et al., 2014a). Finally, the reduction of inflammatory immune responses caused by the downregulation of aegyptin protein would benefit the establishment of infection within the vertebrate host (Chisenhall et al., 2014b).

## Conclusion and future perspectives

Mosquito-transmitted viruses have extended their reach, exposing a substantial portion of the global population to severe health consequences. The inherent variability of arboviruses poses serious challenges for the development of effective vaccines and treatments. Furthermore, classical management strategies to control mosquito populations have been shown to be inefficient to avoid disease spread. For this reason, biological control strategies have emerged as a promising approach in recent years. The introduction of *Wolbachia* bacteria into mosquito populations is a valuable strategy that has gone through extensive testing, including field studies and ethical approvals. However, it is important to acknowledge potential challenges like its effectiveness in certain regions and the putative emergence of escape mutants. This highlights the need for the development of complementary strategies. A promising one involves the use of genetically modified arbovirus-resistant mosquitoes to replace susceptible populations, ensuring minimal ecological disruption. While the technological capability exists, a remaining drawback is our lack of a complete understanding of the complex arbovirus-mosquito interaction web. Research emphasis has lied on transcriptomic and proteomic changes, which have already underscored the complexity of deciphering infection mechanisms, especially *in vivo*. However, current technology allows the required combination of high-throughput analyses, including transcriptomic, translatomic, epitranscriptomics, and proteomics, with network-based modeling to define the critical nodes driving virus infection establishment and transmission. These findings should be validated with molecular, functional and fitness studies. Since results might be influenced by various factors including mosquito and virus strains, times of infection and experimental and bioinformatic methods, coordination between different research groups will be key for this major effort to be effective. Despite the challenges, the combined efforts of research, collaboration, and innovation have paved the way for a world where pathogen-resistant mosquitoes can now soar toward reality.

We have made every effort to incorporate all available published data, we apologize for any unintentional oversight.

## Author contributions

MP-T: Conceptualization, Writing – original draft, Writing – review and editing, Investigation. JD: Conceptualization, Supervision, Writing – review and editing, Funding acquisition, Project administration, Resources.
